# Quantifying Heavy Metals Sequestration by Sulfate-Reducing Bacteria in an Acid Mine Drainage-Contaminated Natural Wetland

**DOI:** 10.3389/fmicb.2013.00043

**Published:** 2013-03-12

**Authors:** John W. Moreau, John H. Fournelle, Jillian F. Banfield

**Affiliations:** ^1^School of Earth Sciences, University of MelbourneParkville, VIC, Australia; ^2^Department of Geoscience, University of Wisconsin-MadisonMadison, WI, USA; ^3^Department of Earth and Planetary Science, University of California-BerkeleyBerkeley, CA, USA; ^4^Department of Environmental Science, Policy, and Management, University of California-BerkeleyBerkeley, CA, USA

**Keywords:** acid mine drainage, heavy metals, metal-sulfides, wetlands, bioremediation, electron microprobe, bacterial sulfate reduction, sulfate-reducing bacteria

## Abstract

Bioremediation strategies that depend on bacterial sulfate reduction for heavy metals remediation harness the reactivity of these metals with biogenic aqueous sulfide. Quantitative knowledge of the degree to which specific toxic metals are partitioned into various sulfide, oxide, or other phases is important for predicting the long-term mobility of these metals under environmental conditions. Here we report the quantitative partitioning into sedimentary biogenic sulfides of a suite of metals and metalloids associated with acid mine drainage contamination of a natural estuarine wetland for over a century.

## Introduction

A dynamic interplay of physical, chemical, and biological processes controls long-term metals speciation in wetlands (e.g., Wood and Shelley, 1999; Toevs et al., 2006). Wetlands are known to act as filters for a number of aqueous inorganic and organic contaminants, and can efficiently remove dissolved or colloidal metals from contaminated groundwater (Scholz and Lee, 2005). Diagenetic and biogeochemical reactions essentially control the mobility of metals through wetland sediments via mineral precipitation and dissolution reactions occurring along redox gradients. Sedimentary solid-phase sulfides and oxides, as well as clay minerals, all play important roles in the immobilization of metals. Many empirical studies have observed the removal of dissolved metals by biogenic sulfides, but few have evaluated the net sequestration of metals in a natural system contaminated continuously for a long period of time. Quantitative assessment of metal partitioning in contaminated wetlands will allow for the evaluation of long-term remediation efficiency, and illustrate which minerals play more or less significant roles in metals sequestration. The distribution and stability of heavy metals in contaminated estuarine wetlands will impact carbon cycling and the health of food webs in the so-called “critical zone” for coastal nutrient cycling.

Sequential extraction methods to assess metal contents in wetland sediments have been criticized (e.g., Toevs et al., 2006) as being poorly representative of how different metals can partition into either minerals or organic matter. Hansel et al. (2001), for example, showed that wetland plants can accumulate a significant amount of toxic metals such as arsenic, copper, and lead. In this study, using electron microprobe analysis (EMPA), we quantified the metal(loid) concentrations of authigenic sedimentary sulfide minerals in an acid mine drainage (AMD)-contaminated wetland, to assess the degree of immobilization of arsenic, copper, lead, zinc, cadmium, selenium, and mercury into these minerals. The sulfides were produced *in situ* via bacterial sulfate reduction in wetland sediments, and the particle size, morphology, and aggregation state of these sulfides contrasted sharply with the small amount of non-biogenic pyrite dispersed by tidal activity from processed mine tailings.

## Materials and Methods

### Field site description

**Figure 1:**
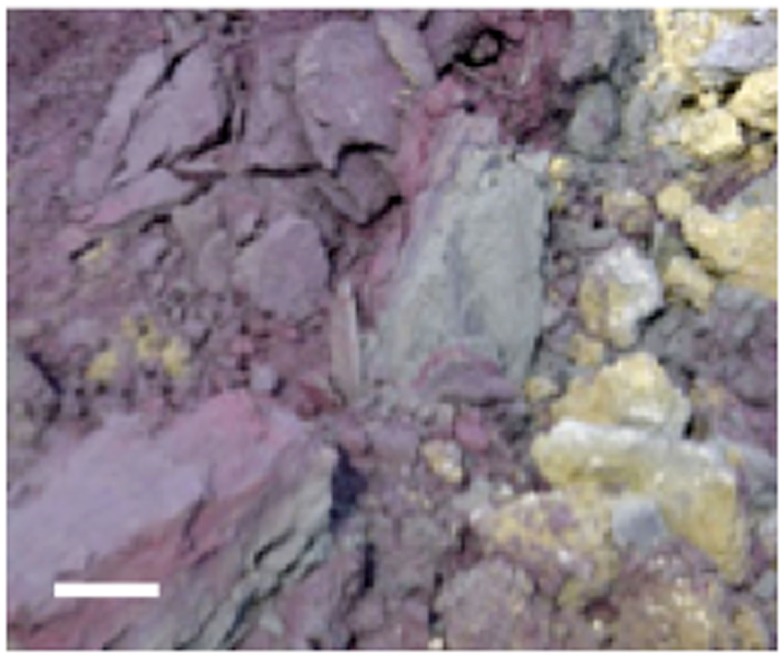
**Photograph of cinder deposits adjacent to acid pond in Stege Marsh**. Purple and gray materials are primarily hematite and specular hematite, and yellowish material is mainly lepidocrocite with some elemental sulfur. Scale bar is ∼10 cm.

Stege Marsh is a natural wetland located along the east central San Francisco Bay, CA, USA. Pyrite “cinders”(pyritic mine tailings heated to high temperatures to leach sulfuric acid) produced by mine tailings processing industries were periodically dumped on nearby tidal flats prior to ∼1950. These cinders (Figure 1), mostly iron oxides such as hematite in composition after processing, were dispersed by tidal action and deposited thinly across Stege Marsh. Surface and near-surface cinders subsequently oxidized to generate AMD for almost a century. Additional mercury fulminate contamination from an explosive blasting cap manufacturing plant nearby also washed into the marsh for some unknown period in the mid-to-late twentieth century. Prior to 2003, the marsh contained a large central AMD pond formed over and adjacent to large buried and surficial cinder deposits. Surrounding this acid pond was a broad acidic zone of reddish-orange and yellow oxidized sediments with sparse or no vegetation. Extending outward from the acid pond were tidal sloughs that traversed the remaining area of Stege Marsh, and allowed for the mixing of AMD generated in the acid pond with brackish tidal waters from the San Francisco Bay.

### Previous studies

URS Corporation was contracted to study the Stege Marsh by the University of California-Berkeley in 2001 (before the timing of this study). Grab samples of sediments were obtained and homogenized at 10 cm intervals from the cinders, acid pond, and upper tidal slough, which were then acid-digested and analyzed via ICP-MS for a suite of metal(loid)s. These data are shown for As, Cd, Cr, Cu, Hg, Pb, Se, and Zn in Table 1, and represent concentrations as ppm (1 wt% = 10^4^ ppm for comparison to the *in situ* data obtained by our study).

**Table 1:** **Whole sediment (acid-digestion) analyses of metals of concern in the Stege Marsh (ppm)**.

	Depth (cm)	pH	As	Cd	Cr	Cu	Hg	Pb	Se	Zn
Cinders (high values^1^)	0	1.8–3.2	220	2200	32	10,000	5300	210	32	23,000
Acid pond	0–10	3.1	1020	2.4	5.1	193	1.3	37.2	17	517
	10–20	4.8	746	3	24	745	27.5	289	53	945
	20–30	6.1	1330	44	ND	1640	166	1240	< 1	5000
Upper slough	0–10	6.9	1400	23	ND	85	3.4	70	ND	300
	10–20	7.6	110	8.5	ND	320	40	72	ND	710
	20–30	7.8	3.9	2.8	ND	15	0.1	6.7	ND	28

*Data were taken from URS Corporation (2002)*.

*^1^URS (2000) Field-sampling and analysis results: Stege Marsh, consultancy report*.

*ND, no data*.

### Field measurements and sampling methods

Field measurements and sampling were performed during low tides. Sampling sites were chosen to represent (1) cinders deposits, (2) sediments impacted directly by the AMD source (“acid pond”), and (3) sediments located proximal to the AMD source in a nearby (but not acidic) major tidal slough that would have been receiving AMD mixed with seawater for a significant period of time (“upper slough”). Measurements of surface or pore water pH were obtained in each site (Table 1) with a field meter/probe (Thermo Orion 5-Star Plus meter with 9107WMMD pH probe).

**Figure 2:**
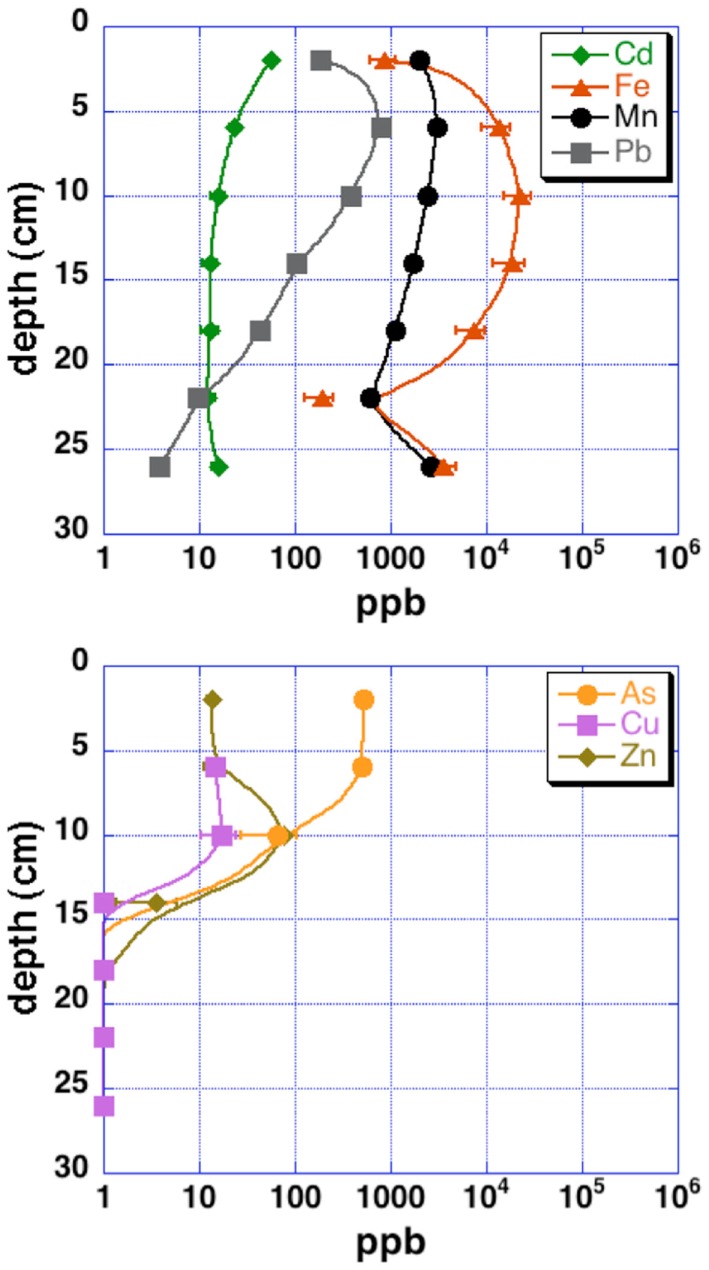
**Pore water metal(loid) concentrations for upper tidal slough sediment cores**. Uncertainties are 2 SD.

Grab samples were taken from cinders upland of the acid pond for mineralogical analysis by X-ray diffraction (XRD). Cores from the upper 30–40 cm of each sampling site (“acid pond” and “tidal slough”) were obtained using a stainless-steel push-coring device. Sleeves were cut to accept sediments up to ∼40 cm deep, but recovered cores were generally slightly shorter. At least three cores were obtained per site, and these were subsampled into 4 and 10 cm intervals, for tidal sloughs and acid pond sediments, respectively, which were subsequently homogenized within these intervals to generate representative materials for EMPA analysis. One lengthwise split of each core was used for a microbial diversity study, as well as for sulfur isotopic analyses of sedimentary metal-sulfides (Moreau et al., 2010). The other split was used to obtain sufficient pore waters via centrifugation for analyses of metal(loid) concentrations (Figure 2).

### Cinder mineralogical analysis

Cinder samples were crushed to a fine powder and resuspended in water. A small amount of cinders was pipetted onto a low-background quartz plate for XRD analysis. XRD was performed on a Bruker-AXS D8 general area detector diffraction system (GADDS) using Co *K*_α_ radiation (1.79026 Å). Two-dimensional diffraction patterns were angle integrated to obtain patterns for analysis. The instrument resolution is 0.07° in 2θ space. All X-ray spectra were processed using the software package EVA (Bruker-AXS).

### Sedimentary metal-sulfide mineralogical analyses

Polished ∼1 cm thick disks of epoxy-impregnated, crushed sediments from each depth of each core were analyzed with EMPA. EMPA was performed with the Cameca SX51 electron probe in the Department of Geoscience, University of Wisconsin-Madison, using wavelength dispersive spectrometry (WDS). Operating conditions were a focused beam at 20 keV and 20 nA, with a 10 s counting time on peak and background positions, with the exceptions of 30 s for As *L*_α_, and 15 s for Zn *K*_α_ and Pb *M*_α_. The probe was operated using Probe for EMPA software for both automation and data reduction (Donovan et al., 2012). The x-ray intensity measurements were corrected with the phi-rho-z matrix correction of Armstrong (1988). Elements, lines and respective crystal assignments used were as follows: TAP, Si *K*_α_, As *L*_α_; PET, S *K*_α_, Cr *K*_α_, Cd *L*_α_ and Pb *M*_α_; LIF, Mn, Fe, Cu, Zn, and Se *K*_α_ and Hg *L*_α_. All reported measurements are >3× the uncertainty of background counts (detection limits).

With regard to interference corrections and pulse height conditions, because of the many higher order interferences, all elements except Zn were acquired with pulse height windows set in differential mode. Peak interference corrections were implemented for interference of As on Pb *M*_α_. Natural and synthetic mineral standards were utilized. Samples were carbon-coated with an approximate thickness of 200 Å, as determined by the interference color on polished brass.

### Measurement of AVS and CRS sedimentary sulfide minerals

Sediment splits from each depth and each core were dried overnight at ∼60°C, and crushed to a fine powder. Between 1–2 g of each powdered sample was processed by the acid-volatile sulfides (AVS) and chromium-reducible sulfides (CRS) extraction/distillation method of Canfield et al. (1986) for monosulfide and disulfide minerals, respectively. Sulfur recovered from each fraction (as Ag_2_S) was weighed in comparison to initial powder weight, to estimate the specific concentration of AVS and CRS sulfides recovered from each depth. A small amount of NIST (http://www.nist.gov/) standard NBS123 Balmat sphalerite was also subjected to this extraction process to test the efficiency of sulfur recovery.

## Results

### Cinder analysis

**Figure 3:**
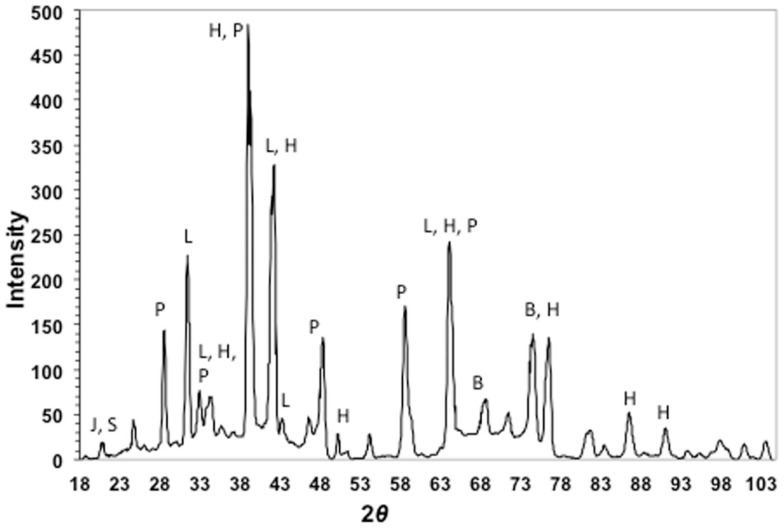
**X-ray diffraction pattern of cinders from Stege Marsh**. J, S, P, L, H, and B stand for jarosite (elemental) sulfur, pyrite, lepidocrocite, hematite, and barite, respectively.

X-ray diffraction patterns of cinders showed a mixture of the iron-sulfoxyhydroxide, iron-hydroxide, and iron-oxide phases jarosite [KFe_3_(SO_4_)_2_(OH)_6_], lepidocrocite (γ-FeOOH), and hematite (Fe_2_O_3_), respectively (Figure 3). Pyrite or marcasite (FeS_2_) was also present, although in minor quantities relative to hematite. Minor elemental sulfur was also detected. The remaining diffraction peaks in the pattern could be assigned to halite and barite. Both of these phases were likely to be present, as they are salts of the two most concentrated seawater anions.

### Sediment core lithologies

**Figure 4:**
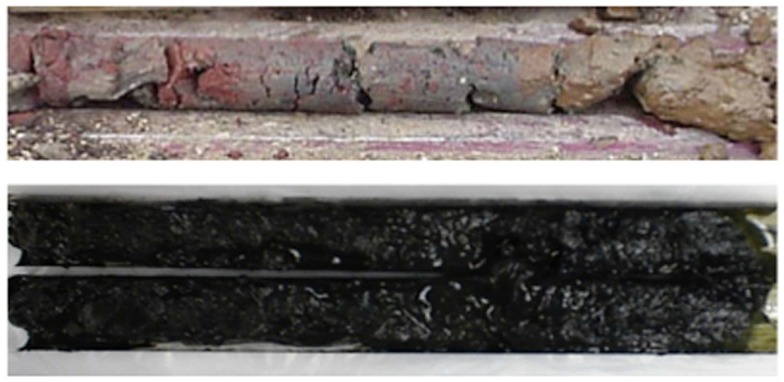
**Representative acid pond and upper tidal slough sediment cores from Stege Marsh**. Acid pond cores (upper) were comprised primarily of reddish hematite and yellowish-brown iron (oxyhydr)oxide. Tidal slough cores (lower) were almost entirely comprised of well-consolidated black muds.

Sediment cores from each sampling site varied greatly in lithology (Figure 4). Some roots, smaller plant detritus, pebbles and gravel, invertebrate shells, and burrows could be identified in the acid pond and tidal slough cores.

Some acid pond cores contained larger stones and cinder aggregates throughout. The uppermost sediments of the acid pond were comprised mainly of interlayered red coarse-grained hematite and gray specular hematite, with intermixed yellowish-brown iron (oxyhydr)oxide phases. Below the surface, the core also contained very small amounts of black fine-grained sulfides. Other acid pond cores contained yellowish iron-oxyhydroxides and elemental sulfur mixed with coarse-grained and specular hematite and fine-grained black iron-sulfides throughout the uppermost 8–10 cm. Below this depth, only coarse-grained and specular hematite was intermixed with sulfides in variable proportions, forming layers that appeared as reddish-brown and purple lenses within the otherwise black sediments.

Tidal slough cores were entirely comprised of well-consolidated black muds that were enriched in iron sulfides (most likely pyrite but possibly some marcasite, see below) below the upper few centimeters. The surface layer of each slough core consisted of a water-saturated greenish-black organic-sediment slurry with streaks of bright yellow elemental sulfur.

### Sedimentary sulfide mineralogy and metals concentrations

**Figure 5:**
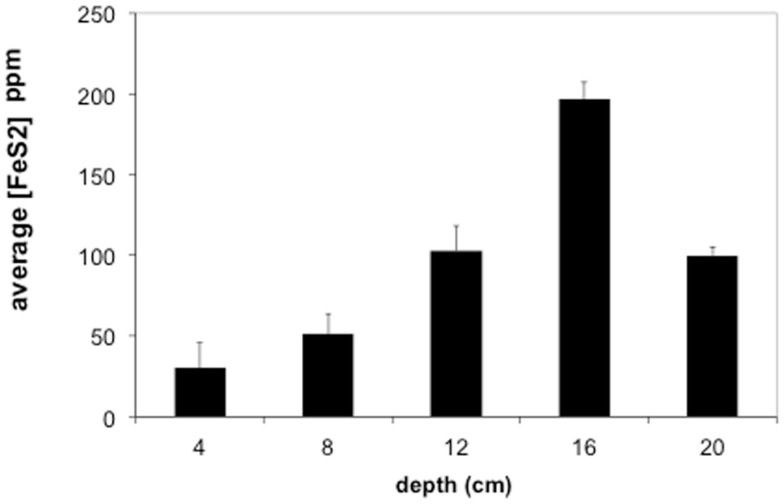
**Average concentrations of pyrite (FeS_2_) in Stege Marsh sediments**. Pyrite concentrations are given as parts per million (ppm). Error bars represent one standard error.

Sediments from the acid pond cores consisted primarily of cinders intermixed with coarse-grained, irregularly shaped iron oxides and sulfides (mostly hematite and FeS_2_, but also some sphalerite), with partly weathered textures. No spheroidal or framboidal metal-sulfides were observed in acid pond core sediments. The concentrations of CRS increased significantly with depth, from ∼5 wt% at 5 cm to a maximum of about 21 wt% at 20 cm (Figure 5). Little (<0.1 wt%) to no AVS was recovered, however.

**Figure 6:**
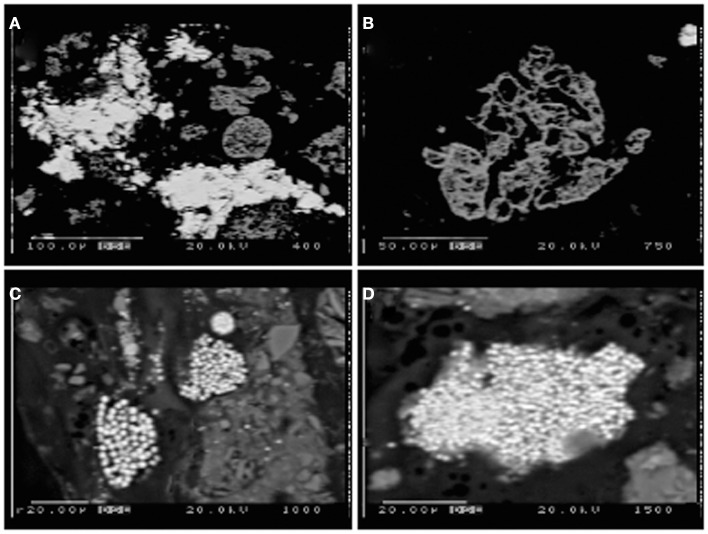
**Electron microprobe images of Stege Marsh sediments**. **(A)** weathered hematite with ZnS infill and/or overgrowth from acid pond sediments, **(B)** weathered hematite in acid pond sediments, **(C,D)** fine-grained FeS_2_ in tidal slough sediments. Scale bars are shown individually in each image.

Tidal slough sediments, in contrast to the acid pond, contained sulfides present mostly as spheroidal and framboidal aggregates of very fine-grained FeS_2_, and abundant iron oxides with textural evidence for vigorous weathering by dissolution and reprecipitation as metal-sulfides (e.g., pitting, extremely fine-grained zinc- and iron-sulfide in-fills and overgrowths; Figures 6A,B). Slough sediments also contained extremely fine-grained AVS, likely iron-monosulfides, as evidenced by the strong smell of sulfide released upon acidification with a few drops of 6N HCl. However, relatively small quantities of AVS were recovered by acid distillation from either slough core at any depth (<2 wt%).

Most of the sulfides present in slough sediments were pyrite or its polymorph marcasite (CRS), as determined by analysis with EMPA (i.e., Fe to S elemental weight percentage ratios of 0.8–0.9). Normally, EMPA analytical totals on large flat polished phases, where all elements are accounted for, should total at least 98–99 wt%. However, here, due to observed presence of abundant organic matter (C was not measured directly) and small sample sizes, analytical totals down to 90 wt% were accepted. In the upper slough, the concentration of CRS increased with depth to a maximum of ∼17 wt% at 16 cm (Figure 5).

### Contaminant metal(loid) distributions in wetland sediments

Sulfur isotopic analyses (Moreau et al., 2010), along with the very fine grain sizes and framboidal or spheroidal character (Figures 6C,D) were used to recognize the biogenic origin of metal-sulfides in slough sediments. These minerals exhibited very different morphological and isotopic properties in comparison to tailings-derived primary sulfides that were present in very small quantities in acid pond sediments. The primary sulfides consisted of larger clasts with no recognizable subgrain particulate structure, whereas biogenic framboids/spheroids clearly consisted of submicron-scale sulfide particles tightly aggregated. However, EMPA scans suggested that most primary sulfides were completely oxidized to hematite during leaching of the sulfur for sulfuric acid manufacturing (e.g., Figure 6B). In some cases, the hematite hosted infill mineralization by zinc-sulfide, which preserved nearly the original shape of the hematite clasts (Figure 6A).

Concentration ranges (as wt%) of contaminant metal(loid)s in authigenic iron and zinc-sulfides are shown in Table 2. Measured concentrations of arsenic, copper, and lead associated with biogenic iron sulfides showed that up to ∼2 wt% of arsenic and copper and ∼0.25 wt% of lead was sequestered into framboidal FeS_2_. The presence of trace zinc in iron sulfides consisted of up to ∼0.5 wt%. Nearly all of the remaining zinc found in the slough sediments was present as a zinc-sulfide phase (probably sphalerite but possibly some biogenic wurtzite; Moreau et al., 2004). Where cadmium was present, it was always observed in association with zinc-sulfide infill (up to ∼0.5 wt%). Interestingly, almost no mercury, chromium, or selenium were observed in association with either biogenic iron or zinc-sulfides. These data may be compared to whole sediment acid-digestion based analyses of Stege Marsh sediments (Table 1) and pore water metal(loid) concentrations (Figure 2) to consider metal(loid) bioremediation efficiency.

**Table 2:** **Concentrations of elements in metal-sulfides by electron microprobe analysis**.

Minimum detection limits	0.02	0.10	0.14	0.02	0.05	0.07	0.10	0.12	0.54	0.21	0.12	0.24	**n/a**
**REFERENCE STANDARD (ELBA PYRITE)**													
841	BDL	**53.27**	BDL	BDL	0.05	**47.28**	BDL	BDL	BDL	BDL	BDL	BDL	**100.60**
842	BDL	**52.88**	BDL	BDL	BDL	**46.75**	BDL	BDL	BDL	BDL	BDL	BDL	**99.63**
843	BDL	**53.25**	BDL	BDL	BDL	**45.95**	BDL	BDL	BDL	BDL	BDL	BDL	**99.19**
**TIDAL SLOUGHS** **0–4 cm, 15 × 15 μm cluster of iron sulfide particles**													
846	0.11	**52.54**	BDL	BDL	BDL	**45.34**	BDL	BDL	BDL	BDL	BDL	**0.25**	**98.25**
847	0.15	**50.91**	BDL	BDL	BDL	**44.54**	BDL	BDL	BDL	BDL	BDL	BDL	**95.60**
**0–4 cm loosely clustered framboidal iron sulfide**													
1446	BDL	**49.91**	BDL	BDL	BDL	**43.08**	BDL	BDL	BDL	BDL	**0.14**	BDL	**93.13**
1449	0.03	**48.92**	BDL	BDL	BDL	**42.96**	BDL	BDL	BDL	BDL	**0.14**	BDL	**92.05**
1450	0.09	**48.65**	BDL	BDL	BDL	**43.02**	BDL	BDL	BDL	BDL	**0.57**	BDL	**92.33**
1451	0.80	**49.54**	BDL	BDL	BDL	**43.66**	BDL	BDL	BDL	BDL	**0.54**	BDL	**94.54**
**4–8 cm 10 μm iron sulfide cluster**													
1212	0.05	**40.68**	BDL	BDL	BDL	**58.40**	BDL	BDL	BDL	BDL	BDL	BDL	**99.13**
1213	BDL	**38.60**	BDL	BDL	BDL	**58.39**	BDL	**0.16**	BDL	BDL	BDL	BDL	**97.15**
1214	0.06	**39.03**	BDL	BDL	BDL	**58.07**	BDL	BDL	BDL	BDL	BDL	BDL	**97.16**
1215	BDL	**39.59**	BDL	BDL	BDL	**58.98**	**0.23**	BDL	BDL	BDL	BDL	BDL	**98.80**
**4–8 cm 6 μm iron sulfide framboids**													
1216	0.45	**47.65**	BDL	**0.02**	0.38	**40.94**	**0.18**	BDL	BDL	BDL	BDL	BDL	**89.61**
**4–8 cm 10 × 10 μm cluster of iron sulfide spheroids**													
1218	0.25	**50.40**	BDL	BDL	0.15	**42.72**	BDL	BDL	BDL	BDL	**0.16**	BDL	**93.68**
1219	0.18	**51.44**	BDL	BDL	0.21	**44.46**	BDL	BDL	BDL	BDL	BDL	BDL	**96.29**
1220	0.16	**50.72**	BDL	BDL	0.09	**45.58**	BDL	BDL	BDL	BDL	BDL	BDL	**96.56**
1221	0.20	**48.74**	BDL	BDL	**0.15**	**44.70**	BDL	BDL	BDL	BDL	BDL	BDL	**93.79**
**4–8 cm 5 μm iron sulfide framboids**													
1222	0.24	**49.93**	BDL	BDL	**0.18**	**44.00**	**0.21**	BDL	BDL	BDL	**0.24**	BDL	**94.81**
**4–8 cm 2 μm iron sulfide framboids, scattered**													
1224	1.40	**47.09**	BDL	BDL	**1.02**	**40.71**	BDL	BDL	BDL	BDL	BDL	BDL	**90.22**
**8–12 cm 5 μm framboidal iron sulfide**													
850	0.09	**51.27**	BDL	**0.02**	**0.08**	**45.30**	BDL	BDL	BDL	BDL	**0.51**	BDL	**97.26**
**8–12 cm 10 μm framboidal iron sulfide**													
851	0.18	**48.21**	BDL	BDL	**0.31**	**44.55**	BDL	BDL	BDL	BDL	**0.24**	BDL	**93.49**
**8–12 cm 5 μm framboidal iron sulfide**													
853	2.66	**50.71**	BDL	BDL	**0.06**	**43.02**	BDL	BDL	BDL	BDL	BDL	BDL	**96.44**
**8–12 cm 5 μm framboidal iron sulfide**													
856	0.50	**46.90**	BDL	BDL	**0.09**	**42.03**	BDL	BDL	BDL	**0.23**	**0.92**	BDL	**90.67**
**8–12 cm 5 μm framboidal iron sulfide**													
859	0.35	**50.68**	BDL	BDL	**0.07**	**43.74**	0.15	BDL	BDL	BDL	BDL	BDL	**94.99**
**8–12 cm 5 μm iron sulfide**													
860	0.27	**49.47**	BDL	BDL	BDL	**44.75**	BDL	BDL	BDL	BDL	BDL	BDL	**94.50**
**8–12 cm 5 μm iron sulfide**													
862	0.07	**50.01**	BDL	BDL	**0.44**	**42.97**	**0.14**	BDL	BDL	BDL	BDL	BDL	**93.63**
**8–12 cm cluster of 2–10 μm iron sulfide framboids**													
863	2.39	**50.49**	BDL	BDL	**0.05**	**43.74**	BDL	BDL	BDL	BDL	**0.60**	BDL	**97.28**
**8–12 cm cluster of 2–10 μm iron sulfide framboids**													
864	1.46	**50.89**	BDL	BDL	**0.06**	**42.83**	**0.13**	BDL	BDL	BDL	**0.39**	BDL	**95.75**
**8–12 cm cluster of 2–10 μm iron sulfide framboids**													
866	1.92	**47.56**	BDL	**0.02**	**0.11**	**40.68**	BDL	BDL	BDL	BDL	**1.62**	BDL	**91.92**
**8–12 cm pair Of 5–7 μm iron sulfide framboids**													
867	1.00	**47.88**	BDL	BDL	**0.69**	**42.03**	BDL	BDL	BDL	BDL	BDL	BDL	**91.60**
**8–12 cm small cluster of 10 μm sulfide framboids**													
868	0.93	**51.15**	BDL	BDL	**0.21**	**44.83**	BDL	BDL	BDL	BDL	BDL	BDL	**97.12**
**8–12 cm small cluster of 10 μm sulfide framboids**													
869	2.68	**46.43**	BDL	BDL	**0.20**	**41.82**	BDL	BDL	BDL	BDL	BDL	BDL	**91.13**
**8–12 cm cluster of 2–10 μm iron sulfide framboids**													
1119	0.51	**50.82**	BDL	BDL	**0.06**	**42.47**	**0.10**	BDL	BDL	BDL	**1.97**	BDL	**95.93**
1121	0.29	**52.18**	BDL	BDL	**0.07**	**46.08**	BDL	BDL	BDL	BDL	**0.54**	BDL	**99.16**
1122	4.20	**47.81**	BDL	BDL	**0.09**	**38.80**	BDL	BDL	BDL	BDL	**1.42**	BDL	**92.33**
1123	1.04	**48.70**	BDL	BDL	**0.11**	**41.81**	BDL	BDL	BDL	BDL	**1.80**	BDL	**93.47**
1124	0.60	**48.17**	BDL	BDL	**0.09**	**42.71**	**0.12**	BDL	BDL	BDL	**1.27**	BDL	**92.97**
1125	1.55	**46.26**	BDL	BDL	**0.08**	**40.03**	**0.11**	BDL	BDL	BDL	**1.90**	BDL	**89.93**
1127	0.63	**51.43**	BDL	BDL	**0.06**	**44.05**	BDL	BDL	**0.29**	BDL	**0.23**	**0.24**	**96.93**
1128	7.79	**45.62**	BDL	**0.03**	**0.12**	**37.85**	BDL	BDL	BDL	BDL	**0.48**	BDL	**91.89**
1129	4.59	**47.44**	BDL	BDL	**0.07**	**41.07**	BDL	BDL	BDL	BDL	**1.59**	BDL	**94.75**
1130	5.45	**45.46**	BDL	BDL	**0.13**	**38.82**	**0.12**	BDL	BDL	BDL	**1.57**	BDL	**91.54**
1131	2.76	**49.47**	BDL	**0.02**	**0.09**	**42.03**	BDL	BDL	BDL	BDL	**0.36**	BDL	**94.72**
**8–12 cm cluster of iron sulfide framboids**													
1132	0.50	**51.81**	BDL	BDL	**0.08**	**44.61**	BDL	BDL	BDL	BDL	BDL	BDL	**97.01**
1133	0.45	**48.15**	BDL	BDL	**0.21**	**42.37**	BDL	BDL	BDL	BDL	BDL	BDL	**91.18**
1137	0.48	**51.09**	BDL	BDL	**0.11**	**44.25**	BDL	BDL	BDL	**0.21**	BDL	BDL	**96.13**
**8–12 cm cluster of 2–10 μm iron sulfide framboids**													
1138	0.38	**49.94**	BDL	BDL	BDL	**42.63**	BDL	BDL	BDL	BDL	BDL	BDL	**92.95**
1140	0.09	**50.90**	BDL	BDL	**0.08**	**43.94**	**0.11**	BDL	BDL	BDL	BDL	BDL	**95.12**
1141	0.25	**51.60**	BDL	BDL	**0.05**	**45.41**	BDL	BDL	BDL	BDL	BDL	BDL	**97.31**
1142	0.17	**49.97**	BDL	BDL	**0.05**	**44.46**	BDL	BDL	BDL	BDL	BDL	BDL	**94.65**
**8–12 cm 5 μm iron sulfide spheroid**													
1147	0.28	**49.80**	BDL	BDL	**0.11**	**43.11**	**0.46**	BDL	BDL	BDL	**0.45**	BDL	**94.21**
**8–12 cm 20 × 15 ellipsoidal iron sulfide**													
1336	0.06	**52.49**	BDL	BDL	BDL	**46.39**	BDL	BDL	BDL	BDL	BDL	**0.24**	**99.18**
**8–12 cm 10 μm** **ellipsoidal iron sulfide**													
1337	0.15	**46.53**	BDL	BDL	BDL	**46.88**	BDL	BDL	BDL	BDL	**0.45**	BDL	**94.01**
**8–12 cm 25 μm spheroidal iron sulfides**													
1338	0.10	**49.48**	BDL	BDL	0.03	**45.06**	BDL	BDL	BDL	BDL	**0.41**	BDL	**95.08**
**8–12 cm 25 μm spheroidal iron sulfide**													
1340	0.07	**50.10**	BDL	**0.03**	BDL	**45.35**	BDL	BDL	BDL	BDL	**0.26**	BDL	**95.82**
1341	0.03	**50.70**	BDL	BDL	BDL	**45.13**	BDL	**0.12**	BDL	BDL	**0.26**	**0.28**	**96.52**
1342	0.03	**51.05**	BDL	**0.03**	BDL	**44.19**	BDL	BDL	BDL	BDL	**0.33**	BDL	**95.62**
**12–16 cm 20 μm iron sulfide aggregate**													
1151	0.03	**40.11**	BDL	BDL	BDL	**58.09**	**0.14**	BDL	BDL	BDL	BDL	BDL	**98.37**
**12–16 cm 6 μm iron sulfide spheroid**													
1153	0.34	**50.42**	BDL	BDL	**0.79**	**43.56**	BDL	BDL	BDL	BDL	BDL	BDL	**95.11**
**12–16 cm 20 μm iron sulfide spheroid**													
1154	0.06	**52.57**	BDL	BDL	**0.83**	**44.69**	BDL	BDL	BDL	BDL	BDL	BDL	**98.14**
1155	0.03	**52.97**	BDL	BDL	**0.74**	**44.06**	BDL	BDL	BDL	BDL	BDL	BDL	**97.80**
1156	BDL	**50.72**	BDL	BDL	**0.79**	**43.54**	BDL	BDL	BDL	BDL	BDL	BDL	**95.05**
1157	0.09	**50.75**	BDL	BDL	**0.80**	**43.82**	BDL	BDL	BDL	BDL	BDL	BDL	**95.46**
1158	0.04	**48.22**	BDL	BDL	**0.83**	**44.01**	BDL	BDL	BDL	BDL	BDL	BDL	**93.11**
**12–16 cm cluster of 1 μm iron sulfides**													
1159	0.95	**33.27**	BDL	BDL	BDL	**21.44**	**31.41**	**0.50**	BDL	BDL	**0.93**	**0.27**	**88.77**
**12–16 cm 5 μm sulfide spheroid**													
1173	0.77	**48.22**	BDL	BDL	**0.06**	**43.80**	BDL	BDL	BDL	BDL	BDL	BDL	**92.84**
**12–16 cm 5 μm sulfide spheroid**													
1174	1.20	**48.90**	BDL	BDL	**0.05**	**42.05**	**0.50**	BDL	BDL	BDL	BDL	BDL	**92.70**
**16–20 cm cluster of 5 μm iron sulfide spheroids**													
1177	0.19	**52.11**	BDL	BDL	BDL	**46.15**	BDL	BDL	BDL	BDL	BDL	**0.26**	**98.71**
1179	0.47	**48.28**	BDL	BDL	BDL	**44.79**	BDL	BDL	BDL	BDL	**0.14**	BDL	**93.68**
1180	0.26	**45.60**	BDL	BDL	BDL	**44.24**	BDL	BDL	BDL	BDL	BDL	BDL	**90.10**
**16–20 cm 12 μm iron sulfide framboid**													
1182	0.49	**50.79**	BDL	BDL	BDL	**45.27**	BDL	BDL	BDL	BDL	**0.40**	BDL	**96.95**
**16–20 cm 13 μm iron sulfide framboid**													
1189	0.19	**49.04**	BDL	BDL	BDL	**45.41**	BDL	BDL	BDL	BDL	**0.20**	BDL	**94.85**
**16–20 cm 16 μm iron sulfide framboids**													
1437	0.34	**52.06**	BDL	BDL	BDL	**46.77**	**0.12**	BDL	BDL	BDL	**0.17**	BDL	**99.47**
1438	0.18	**51.58**	BDL	**0.02**	BDL	**46.10**	**0.14**	BDL	BDL	BDL	**0.15**	BDL	**98.16**
**16–20 cm 15 μm iron sulfide framboids**													
1439	0.08	**47.90**	BDL	BDL	**0.11**	**42.80**	BDL	BDL	BDL	BDL	BDL	BDL	**90.89**
1440	0.21	**49.31**	BDL	BDL	**0.12**	**43.50**	**0.14**	BDL	BDL	BDL	BDL	BDL	**93.28**
**16–20 cm 15 μm iron sulfide framboids**													
1441	0.08	**48.28**	BDL	**0.02**	BDL	**45.60**	**0.09**	BDL	BDL	BDL	BDL	BDL	**94.07**
1442	0.27	**51.65**	BDL	BDL	**0.07**	**45.41**	BDL	BDL	BDL	BDL	BDL	BDL	**97.41**
**16–20 cm 20 μm iron sulfide framboids**													
1443	0.16	**51.42**	BDL	BDL	BDL	**45.51**	BDL	BDL	BDL	BDL	BDL	BDL	**97.09**
**20–24 cm 18 μm iron sulfide framboids**													
1398	0.26	**52.74**	BDL	BDL	BDL	**45.10**	BDL	BDL	BDL	BDL	BDL	BDL	**98.09**
1399	0.11	**54.05**	BDL	BDL	BDL	**46.22**	BDL	BDL	BDL	BDL	BDL	BDL	**100.38**
**20–24 cm 5 μm iron sulfide spheroids**													
1403	0.13	**50.12**	BDL	BDL	**0.43**	**44.66**	BDL	BDL	BDL	BDL	BDL	BDL	**95.33**
**20–24 cm 25 × 15 μm iron sulfide**													
1405	0.05	**50.85**	BDL	BDL	BDL	**44.14**	**0.18**	BDL	BDL	BDL	**0.69**	BDL	**95.91**
**20–24 cm 95 μm iron sulfide framboid**													
1406	0.42	**44.75**	BDL	BDL	BDL	**45.74**	**0.10**	BDL	BDL	BDL	**0.78**	BDL	**91.79**
**24–28 cm zinc-sulfide infilling in weathered iron oxide**													
1194	BDL	**32.36**	**0.47**	BDL	BDL	**1.82**	**0.32**	**63.26**	BDL	BDL	BDL	BDL	**98.22**
1195	BDL	**33.04**	**0.46**	BDL	BDL	**2.17**	**0.41**	**64.16**	BDL	BDL	BDL	BDL	**100.25**
**24–28 cm zinc-sulfide 10 μm aggregate**													
1196	0.04	**33.17**	**0.38**	BDL	BDL	**3.74**	**0.76**	**59.01**	BDL	BDL	BDL	BDL	**97.10**
1197	0.12	**33.34**	**0.36**	BDL	BDL	**4.04**	**0.52**	**59.83**	BDL	BDL	BDL	BDL	**98.22**
**24–28 cm iron sulfide 10 μm spheroid**													
1198	0.09	**37.40**	BDL	BDL	BDL	**57.11**	**0.42**	BDL	BDL	BDL	BDL	BDL	**95.01**
**24–28 cm 60 × 60 μm region of iron sulfide overgrowths**													
1202	0.03	**37.75**	BDL	**0.02**	BDL	**57.67**	**2.04**	BDL	BDL	BDL	BDL	BDL	**97.51**
**24–28 cm 60 × 60 μm region of iron sulfide overgrowths**													
1204	0.03	**37.93**	BDL	BDL	BDL	**57.16**	**1.08**	BDL	BDL	BDL	BDL	BDL	**96.20**
1205	BDL	**37.53**	BDL	**0.02**	BDL	**57.58**	**0.85**	BDL	BDL	BDL	BDL	**0.24**	**96.21**
1206	0.03	**37.10**	BDL	BDL	BDL	**57.35**	**1.56**	BDL	BDL	BDL	BDL	BDL	**96.04**
**24–28 cm 8 μm long zinc-sulfide replacement on iron oxide**													
1207	0.19	**31.11**	**0.37**	BDL	BDL	**5.15**	**0.41**	**58.25**	BDL	BDL	BDL	BDL	**95.48**
**24–28 cm 8 × 8 μm zinc-sulfide replacement within fe-oxide**													
1208	0.08	**31.08**	**0.42**	BDL	BDL	**2.92**	**0.11**	**62.52**	BDL	BDL	BDL	BDL	**97.13**

*Concentrations are given as wt% of iron- or zinc-sulfides selected for *in situ* analyses. Bold numbers indicate measurements that exceeded the minimum detection limit (>3 SD of the background counts). Only analyses with total wt% >90 were used*.

## Discussion

The contaminant metals of interest were almost exclusively contained within iron sulfide grains or aggregates. The distribution of iron sulfides reaches a maximum (∼200 ppm whole sediments) at 12–16 cm below the surface in the tidal sloughs, with lesser but significant amounts (∼100 ppm) at 8–12 and 16–20 cm depth intervals. We interpret this depth of locally increased iron sulfide concentrations to reflect the primary zone for bacterial sulfate reduction in wetland sediments, where biogenic (aqueous) sulfide reacts with ferrous iron and dissolved zinc to form FeS_2_ and ZnS, respectively. This interpretation is consistent with previously published geochemical and isotopic profiles of pore water sulfate and sedimentary sulfides from this study site (Moreau et al., 2010). The highest concentrations of As, Cu, and Pb measured with EMPA in biogenic FeS_2_ aggregates were greater than those measured in whole sediment digestions by ∼14, 63, and 35 times, respectively, illustrating the efficiency of bacterial sulfate reduction in sequestering these metal(loid)s into biogenic FeS_2_. By comparison, the highest concentrations of Cd measured with EMPA in authigenic ZnS were only ∼7 times higher than Cd concentrations measured in whole sediment digestions. These findings imply that the bioremediation efficiency of some metal(loid)s (e.g., As, Cu, Pb) will depend more on the availability of aqueous Fe^2+^ to react with biogenic HS^−^ to precipitate Fe(As, Cu, Pb)S_2_, while metals like Cd seem to depend more on the availability of dissolved Zn to precipitate as Zn(Cd_1−Zn_)S. Comparison of EMPA results to sediment pore water metal(loid) concentrations for the upper tidal slough (Figure 2) supports this interpretation based on the relative greater abundance of dissolved Fe^2+^ over Zn^2+^.

Our observations suggest that the first 0–8 cm and below 20–24 cm depths in AMD-impacted wetlands may not efficiently sequester metals into sedimentary sulfide minerals. Presumably, conditions are insufficiently reducing for extended periods of time nearer the sediment-seawater interface, where sediments are under more acidic conditions than non-contaminated tidal wetlands (e.g., Moreau et al., 2010). At depths below ∼20 cm, conditions may be unfavorable for sulfate-reducing bacteria to thrive (e.g., reduced sulfate concentrations). Comparison of EMPA results with pore water metal(loid) concentrations in the upper tidal slough confirms this interpretation by showing how As, Cu, Zn, and Cd concentrations all achieve their minima at ∼16 cm below surface, while Fe^2+^ levels begin to drop at roughly this depth. Note that As, Cu, Cd, Pb, and Zn all decrease to below pore water regulatory levels by ∼15 cm depth, or 20–25 cm in the case of lead, possibly consistent with a delayed onset for galena precipitation (Druschel et al., 2002). Manganese and lead offer less clear indications of depth dependency, but we note that both metals’ concentrations were decreasing by the 12–16 cm depth interval in upper tidal slough sediments. Unfortunately, Hg, Se, and Cr were generally below detection limits and thus no mineralogical or depth-dependent trends could be determined on the basis of our existing data. Thus, the natural wetland under long-term AMD contamination in this study revealed that wetlands constructed for similar purposes may exhibit depth-dependent variability in efficiency of contaminant removal, particularly in the case of heavy metals removed as sulfides or sulfide impurities. This finding has important implications for metals bioavailability and toxicity to sediment microorganisms and their ability to degrade organic carbon in the critical zone, as well as for the invertebrates that graze on these microorganisms and their predation in turn by macrofauna (e.g., wetland birds; Levin et al., 2001).

Backscattered imaging data provided abundant evidence for highly weathered textures of iron oxides, which presumably provided the ferrous iron for localized FeS_2_. We note that iron ox(yhydrox)ides may be dissolved either directly (i.e., by iron-reducing bacteria) or indirectly (e.g., coupled with the oxidation of aqueous sulfide). In this case, the close association of ZnS with highly weathered iron oxides, as opposed to as spatially distinct spheroidal ZnS (e.g., Moreau et al., 2007), suggests the mechanism of indirect reductive dissolution may provide a more plausible explanation. Previous work has shown that zinc accumulates in association with iron oxides in mine tailings under acidic oxidizing conditions, either as an adsorbed cation initially or as goslarite (ZnSO_4_-7H_2_O) after prolonged acid weathering (Schuwirth et al., 2007; Hayes et al., 2011). We speculate that zinc may have been concentrated on the hematite when the tailings from ore processing were first deposited in Stege Marsh, under subaerial conditions. This zinc would then have reacted, under flooded reducing conditions, with biogenic sulfide to form the “infilling” ZnS. Notably, few to no colloidal or framboidal aggregates of ZnS were observed, although textural evidence from EMPA images of ZnS infill suggests a very fine-grained (i.e., nanoparticulate) character. Instead, nearly all framboidal or spheroidal aggregates were composed of FeS_2_, suggesting rapid onset of bacterial sulfate reduction when seawater is present and biogenic (bi)sulfide concentrations well in excess of dissolved metals. No evidence for sequential precipitation (e.g., overgrowths) or spatial separation of metal-sulfide phases (e.g., Druschel et al., 2002) was observed.

The absence of mercury in all but one sample of FeS_2_ (0.29 wt%) is consistent with previous findings that Hg preferentially partitions into natural organic matter, NOM (Reddy and Aiken, 2001; Ravichandran, 2004), in some cases as nanocolloidal metacinnabar (Gerbig et al., 2011). Thus wetland-based strategies for bioremediation of aqueous mercury will need to consider the implications of this process. Recent work has demonstrated the redox reactive nature of NOM-hosted Hg (Zheng et al., 2012), and some research suggests that NOM-bound Hg may be more available for uptake and methylation by bacteria (Graham et al., 2012), even as nanoparticulate HgS (Zhang et al., 2012). However, we note that no nanoparticulate or colloidal HgS was observed in our samples by EMPA.

Selenium can form the anions selenate or selenite, which can remain dissolved unless removed as elemental Se by selenium-reducing bacteria. Although studies have reported Se immobilization by microorganisms in wetlands (e.g., de Souza et al., 1999), no evidence for precipitation of solid-phase Se, either as elemental selenium or within sulfide or oxide minerals, was observed in our tidal slough sediments.

The absence of detectable chromium is consistent with the lack of evidence for precipitation of chromium-oxide phases, presumably due to the preferential leaching of chromate, CrO_4_^2−^, from oxidized tailings (Railsback, 2003). We infer that chromium was more mobile than the other metals due to the absence of Cr-oxides as well as the presence of textural evidence for pervasive reductive dissolution of iron oxide surfaces that could have otherwise served as an adsorbent for chromate (Ajouyed et al., 2010).

In summary, the exposure of Stege Marsh to almost a century of AMD provided the opportunity to study the long-term fate of contaminant metals in sediments, and to evaluate quantitatively the degree and character of heavy metals sequestration into biogenic minerals in a natural model for constructed wetlands. From our observations, we conclude that one-sixth to one-seventh (∼4 cm depth interval) of the tidal slough sediments receiving AMD removed the most metals. Based on EMPA and previous sulfur isotopic analysis (Moreau et al., 2010), this portion of the slough sediments hosts the most active “zone” of sulfate-reducing bacteria, and thus controls metals remediation. As, Cu, and Pb preferentially partitioned into iron sulfides where as Cd was removed within zinc-sulfides. Hg, Se, and Cr were removed poorly, or not at all, by biogenic sulfide mineral formation. The variability in metal sequestration efficiency, directly measured here using *in situ* analysis of biogenic sulfide compositions, highlights the need to consider the use of multiple and/or intermixed mineral surfaces and structures, or mineral-forming processes (e.g., bacterial sulfate reduction) when designing constructed wetlands for AMD remediation.

## Conflict of Interest Statement

The authors declare that the research was conducted in the absence of any commercial or financial relationships that could be construed as a potential conflict of interest.
